# Insight into the Role and Evidence of Oxygen Vacancies in Porous Single-Crystalline Oxide to Enhance Catalytic Activity and Durability

**DOI:** 10.34133/research.0233

**Published:** 2023-09-21

**Authors:** Lingting Ye, Jiaming Ma, Jie Zhang, Wen Yin, Yuanguang Xia, Kui Xie

**Affiliations:** ^1^ Key Laboratory of Optoelectronic Materials Chemistry and Physics, Fujian Institute of Research on the Structure of Matter, Chinese Academy of Sciences, Fuzhou, Fujian 350002, China.; ^2^ Fujian Science & Technology Innovation Laboratory for Optoelectronic Information of China, Fuzhou, Fujian 350108, China.; ^3^ Advanced Energy Science and Technology Guangdong Laboratory, 29 Sanxin North Road, Huizhou, Guangdong 116023, China.; ^4^ Spallation Neutron Source Science Center, Dongguan, Guangdong 523803, China.

## Abstract

Introducing and stabilizing oxygen vacancies in oxide catalysts is considered to be a promising strategy for improving catalytic activity and durability. Herein, we quantitatively create oxygen vacancies in the lattice of porous single-crystalline β-Ga_2_O_3_ monoliths by reduction treatments and stabilize them through the long-range ordering of crystal lattice to enhance catalytic activity and durability. The combination analysis of time-of-flight neutron powder diffraction and extended x-ray absorption fine structure discloses that the preferential generation of oxygen vacancy tends to occur at the site of tetrahedral coordination oxygen ions (O_III_ sites), which contributes to the formation of unsaturated Ga–O coordination in the monoclinic phase. The oxygen vacancies are randomly distributed in lattice even though some of them are present in the form of domain defect in the PSC Ga_2_O_3_ monoliths after the reduction treatment. The number of oxygen vacancies in the reduced monoliths gives 2.32 × 10^13^, 2.87 × 10^13^, and 3.45 × 10^13^ mg^−1^ for the Ga_2_O_2.952_, Ga_2_O_2.895_, and Ga_2_O_2.880_, respectively. We therefore demonstrate the exceptionally high C_2_H_4_ selectivity of ~100% at the C_2_H_6_ conversion of ~37% for nonoxidative dehydrogenation of C_2_H_6_ to C_2_H_4_. We further demonstrate the excellent durability even at 620 °C for 240 h of continuous operation.

## Introduction

Oxygen vacancy (*V*_O_)-associated defects are often considered as active sites that are composed of unsaturated coordination structures in oxide catalysts in catalytic reactions. The concentration of *V*_O_ in metal oxides is usually determined by the oxygen deficiency, which is normally dynamic and controlled by the properties of the electron orbitals of cations in the oxides [1–5]. Therefore, the *V*_O_ needs to be uniformly distributed and effectively stabilized in the crystal lattice. Creating *V*_O_ in the catalysts would highly facilitate the catalytic activity, while stabilizing the *V*_O_ would improve the durability of catalysts [6,7].

β-Ga_2_O_3_ is a highly efficient catalyst that is usually used for light alkane dehydrogenation reaction [8,9]. β-Ga_2_O_3_ is a monoclinic *n*-type semiconductor with the space group of C_2_/m and the lattice parameters *a*, *b*, *c*, and *β* of 12.23 Å, 3.04 Å, 5.80 Å, and 103.7°, respectively [10,11]. The unit cell consists of 2 Ga ions and 3 types of O ions that are located in the tetrahedral and octahedral coordination, respectively. Two O ions have a triple coordination, and the third O has a tetrahedral coordination. Due to the unique feature of electron orbital of Ga, the β-Ga_2_O_3_ is chemically and thermally stable especially at reduced temperatures. Reduction treatment produces *V*_O_ in β-Ga_2_O_3_ at a certain temperature, which creates the low-coordinated Ga active sites in the electron-deficient surface. It has been confirmed that these active sites markedly facilitate the enhancement of catalytic activity of nonoxidative C_2_H_6_ dehydrogenation, especially for the C–H activation, but avoid the C–C bond breakage at reduced temperatures [12–14]. C_2_H_4_ is the most widely used block material in the manufacture of polymers and other chemicals [15,16]. Although the β-Ga_2_O_3_ with oxygen deficiency is highly active [17,18], the activity is only observed at the initial stage because the agglomeration of oxygen defects tends to produce amorphous layers at surface that blocks the active site and degrades the catalytic activity in reactions.

Porous single-crystalline β-Ga_2_O_3_ (PSC Ga_2_O_3_) monoliths at the macroscale incorporates the synergistic effect of interconnected pores and single-crystalline skeleton in the porous architectures, which yields both long-range ordered and well-defined structures of the lattice in the materials [19,20]. The interconnected pores facilitate the rapid diffusion of species and provide a large surface area for chemical reactions. The long-range ordering of the lattice offers a unique path for creating and stabilizing the *V*_O_ in lattice in the interconnected single-crystalline skeletons in the porous architecture. Therefore, PSC Ga_2_O_3_ monoliths at the macroscale would combine the long-range ordering of the lattice and the disordering of the interconnected pores, thereby improving the properties of the materials themselves. These structures have inherent mechanical and thermal stability, facilitate the species diffusion through 3-dimensional percolation, and are easy to process into complex structures. The monoliths would therefore have the advantages of high catalytic performance and thermal durability in the catalytic reactions [21].

Herein, we fabricate PSC Ga_2_O_3_ monolith and engineer the oxygen deficiency in lattice to create active sites associated with oxygen vacancies on the well-defined surface in the porous architectures. We further correlate the concentration of *V*_O_ with catalytic performance. As a case study, we demonstrate the exceptionally high performance of nonoxidative dehydrogenation of C_2_H_6_ to C_2_H_4_ with excellent durability during the continuous operation of 240 h.

## Results

**Fig. 1.**
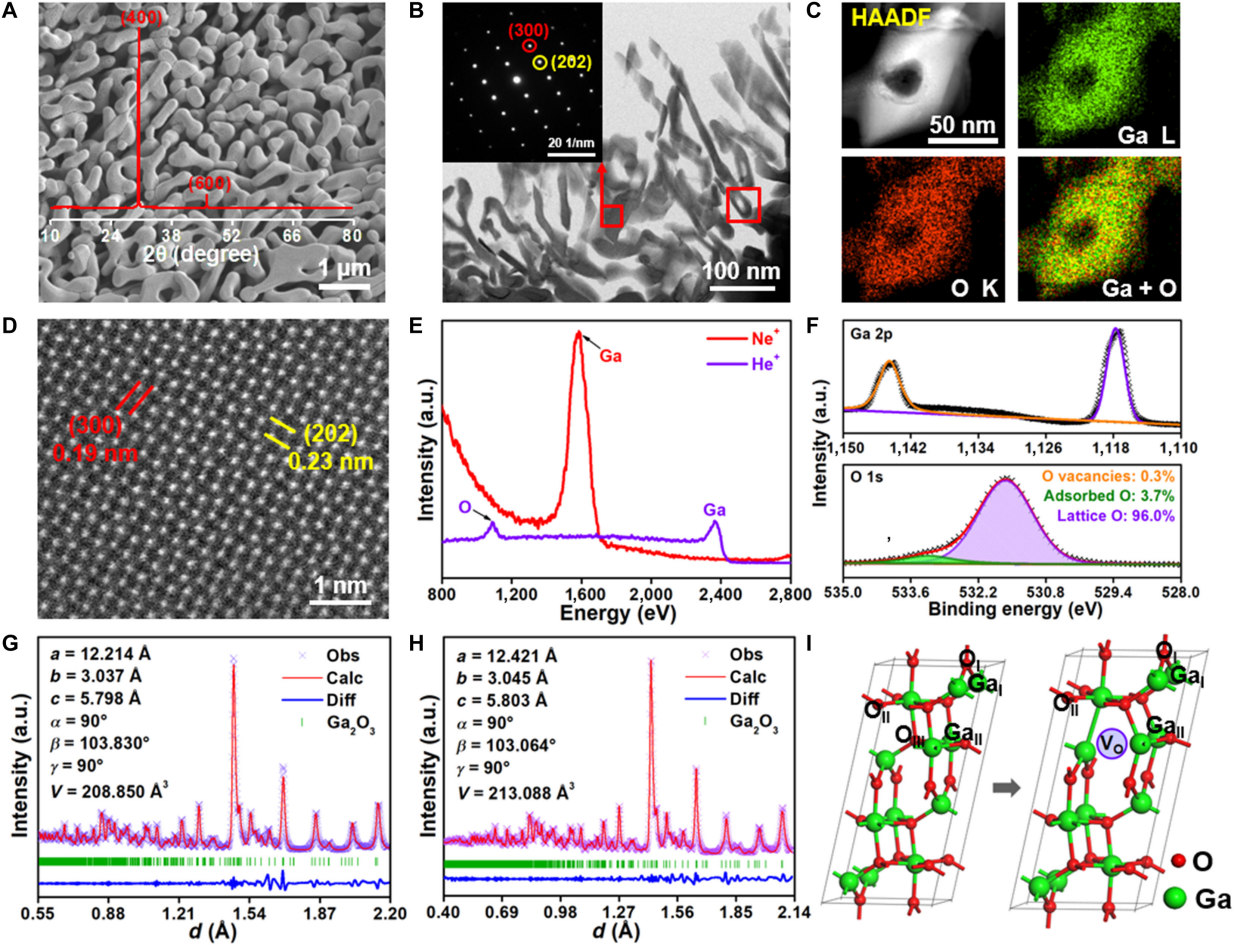
Microstructure of PSC Ga_2_O_3_ monoliths. (A) Scanning electron microscopy and XRD patterns of PSC Ga_2_O_3_ monoliths. (B to D) Scanning transmission electron microscopy, SAED, high-angle annular dark-field (HAADF), element mapping, and Cs-HRTEM of PSC Ga_2_O_3_ monoliths with the face of (100). (E and F) HS-LEISS and XPS of the PSC Ga_2_O_3_ monoliths. (G and H) Observed (crosses), calculated (solid line), and differential TOF NPD patterns of PSC Ga_2_O_3_ powder. (I) Crystal structures of β-Ga_2_O_3_ from TOF NPD datasets. a.u., arbitrary units.

PSC Ga_2_O_3_ monoliths are grown from the mother phase of GaPO_4_ single crystal by a lattice reconstruction strategy in a solid-solid transformation process. The growth mechanism is demonstrated in Figs. S1 and S2, which indicates a lattice channel of P/O atoms. The lattice mismatch between β-Ga_2_O_3_ and GaPO_4_ would dominate the preferential growth of facet in the porous architectures. The porosity is ~66% for PSC Ga_2_O_3_ monoliths, which is favorable for the species diffusion through the 3-dimensional percolation. The x-ray diffraction (XRD) patterns of the PSC Ga_2_O_3_ monoliths with (100) and (001) facets are grown from GaPO_4_ with (−132) and (012) facets, respectively. Figure 1A and Fig. S3 display the microstructures of the PSC Ga_2_O_3_ monoliths. It is shown that the uniform pore size distribution is at ~100 nm. In Fig. S4, Brunauer–Emmett–Teller test further reveals the pore size of ~110 to 130 nm and specific surface area of ~5 m^2^/g. Figure 1B and Fig. S5 show scanning transmission electron microscopy (STEM) and selected area electron diffraction (SAED) images of the PSC Ga_2_O_3_ monoliths, respectively. The porous architecture is pretty uniform with interconnected single-crystalline skeletons. The high-angle annular dark-field image and elemental mappings in Fig. 1C show that the Ga and O are evenly distributed in the skeleton. We have only observed the presence of Ga and O elements in the samples, further proving that the P element is absent, indicating the complete conversion of GaPO_4_ to Ga_2_O_3_ in Fig. S6. The P element has been completely removed during the process by transforming the mother phase of GaPO_4_ single crystal into PSC Ga_2_O_3_ monolith. In addition, the element mapping indicates the absence of element separation in the skeletons. In Fig. 1D, the spherical aberration-corrected high-resolution transmission electron microscopy (Cs-HRTEM) shows the lattice spacings of 0.19 and 0.23 nm, which indicates the good reference to (300) and (202) facets, respectively.

In Fig. 1E and Fig. S7, the high-sensitivity low-energy ion scattering spectra (HS-LEISS) show that the PSC Ga_2_O_3_ monoliths exhibit the presence of Ga and O on the top layer, forming a series of clear Ga–O coordination structures on the surfaces. According to x-ray photoelectron spectroscopy (XPS) in Fig. 1F and Fig. S8, the oxidation states of Ga and O are +3 and −2, respectively. The ratio of O vacancies calculated from the peak area is only 0.3% to 0.4%, which indicates that the O vacancies can be basically ignored in the pristine PSC Ga_2_O_3_ monoliths. Time-of-flight neutron powder diffraction (TOF NPD) in Fig. 1G and H indicates that the PSC Ga_2_O_3_ powder could be indexed assuming a monoclinic with space group C1 2/m. Selected atomic spacings, corresponding to the coordination refined from TOF NPD data, are used to draw the corresponding structure in Fig. 1I. Ga_2_O_3_ has 2 coordination structures, tetrahedral coordination site (Ga(t)) and octahedral one (Ga(o)). In β-Ga_2_O_3_ phase, the ratio of Ga(t) to Ga(o) is known as 1:1 [22,23]. The preferential generation of *V*_O_ tends to occur in the tetrahedral coordination oxygen ions (O_III_ sites), which contributes to the unsaturated Ga–O coordination in the monoclinic phase. This suggests that the active sites tend to be produced at the Ga(t) and further indicates that the catalytic activity of oxygen vacancies located in Ga(t) is superior to that in Ga(o).

**Fig. 2.**
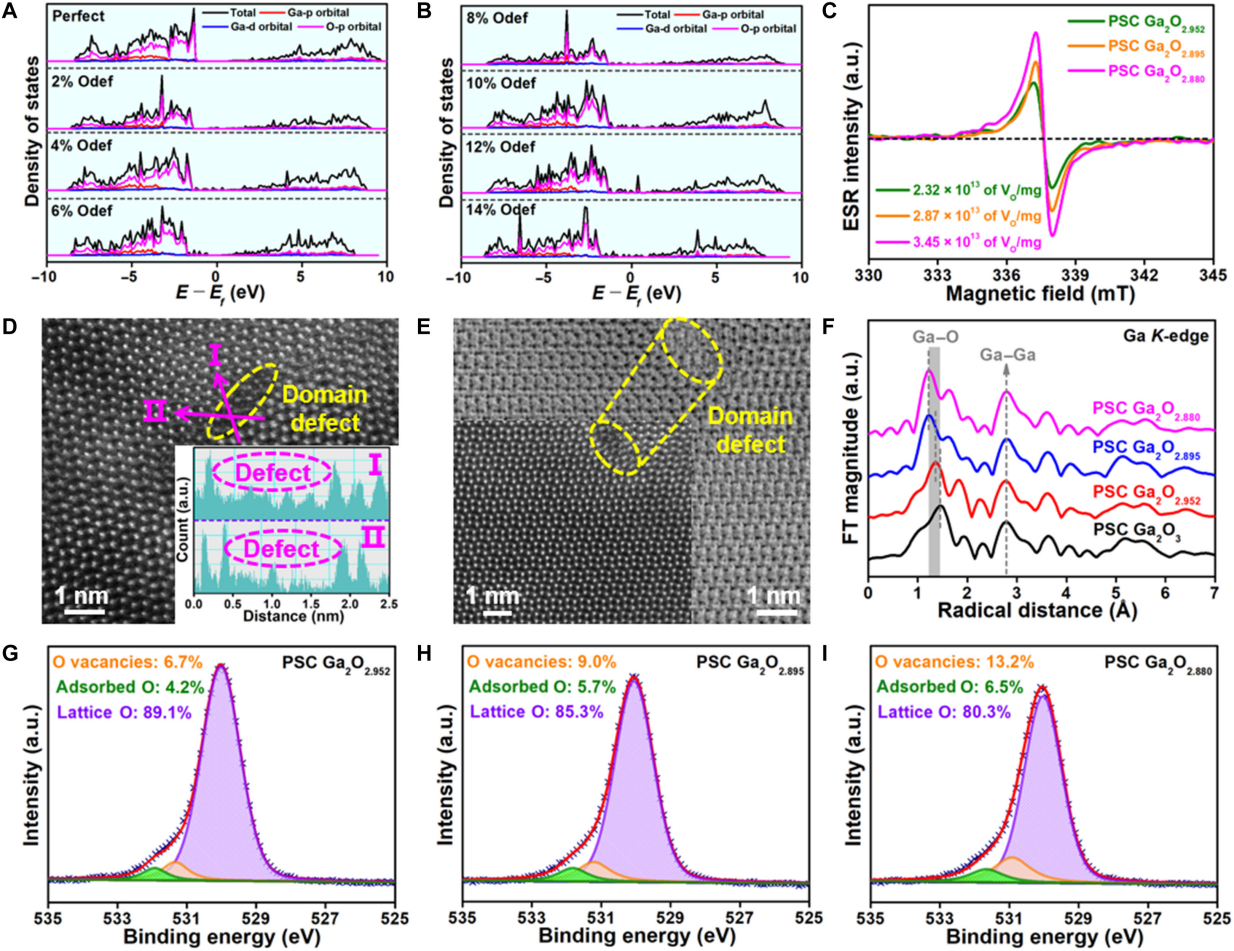
Oxygen vacancies in PSC Ga_2_O_3_ monoliths. (A and B) Density of states of β-Ga_2_O_3_ with different concentrations of *V*_O_s. (C) ESR spectra of the 3 reduced PSC Ga_2_O_3_ monoliths. (D and E) Cs-HRTEM images with different area of the PSC Ga_2_O_2.880_ monoliths. (F) Fourier transforms (FT) derived from EXAFS oscillations with different samples. (G to I) XPS of O 1s in the 3 reduced PSC Ga_2_O_3_ monoliths.

Figure 2A and B and Figs. S9 and S10 show the formation of different concentration of *V*_O_ in lattice, which indicates that the *V*_O_ can be isolated even at the concentration of as high as up to 14%. We show detailed information on the density of states of β-Ga_2_O_3_ with different *V*_O_ concentrations. We can observe that the valence band is mainly made up of the O 2p state with a small amount of the Ga 4p state. The local electronic structure of β-Ga_2_O_3_ also changes with the emergence of *V*_O_ [24]. Moreover, the main changes occur in the valence state of oxygen 2p state, and the band gap width of β-Ga_2_O_3_ with different *V*_O_ concentrations becomes narrower compared to pure β-Ga_2_O_3_. We create the different concentrations of *V*_O_ in lattice by directly reducing the PSC Ga_2_O_3_ monoliths and oxidize the reduced samples by using thermal gravimetric analysis to determine their chemical formula to be Ga_2_O_2.952_, Ga_2_O_2.895_, and Ga_2_O_2.880_, respectively, in Fig. S11. We observe that the XRD of PSC Ga_2_O_3_ monoliths are slightly shifted to a low angle after reduction, indicating that the introduction of a large number of oxygen vacancies may cause the lattice expansion in Fig. S12A. Nevertheless, no phase transition is observed in the β-Ga_2_O_3_ even after the high temperature treatment in a reducing gas, firmly verifying the excellent thermal stability of the PSC Ga_2_O_3_ monoliths. The SAED image of PSC Ga_2_O_2.880_ monoliths reveals that it is a monoclinic structured β-Ga_2_O_3_ single crystal with the (100) facet in Fig. S12B, which is consistent with the XRD result. HS-LEISS is used to identify the ratio between O and Ga at the top layer in Fig. S13, which further confirms the changes in surface oxygen concentration from ~25.3% to ~24.8%. Electron spin resonance (ESR) results as shown in Fig. 2C and Fig. S14 further confirm the existence of *V*_O_. The 3 reduced PSC Ga_2_O_3_ monoliths all show pairs of steep peaks and symmetric distributions at *g* = 2.003, indicating that electrons are trapped in the *V*_O_ [25]. In addition, we further quantify the number of *V*_O_ in the reduced PSC Ga_2_O_3_ monoliths, which gives 2.32 × 10^13^, 2.87 × 10^13^, and 3.45 × 10^13^ mg^−1^ for the Ga_2_O_2.952_, Ga_2_O_2.895_, and Ga_2_O_2.880_, respectively.

Indirect evidence in Cs-HRTEM is usually utilized to assist the proof of the presence of *V*_O_s especially in domain defects in oxide samples [25,26]. We tentatively conduct the structural analysis based on Cs-HRTEM image to visualize possible defects induced in the samples. The disordered Ga atoms in the form of bright dots in the domain in the size of ~1 to 2 nm have been observed in the bright-field Cs-HRTEM image in Fig. 2D with the inset as marked with an ellipse in the line profile. This may be mainly attributed to the loss of oxygen in lattice that contributes to the formation of domain defects. In contrast, the disordered white clusters that represent O atoms in the domain in the size of ~1 to 2 nm have been observed in the dark-field Cs-HRTEM image in Fig. 2E, which is possibly attributed to the loss of lattice oxygen in the sample, whereas the corresponding domain as marked with an ellipse in the dark-field Cs-HRTEM image in Fig. 2E possesses disordered Ga atoms possibly blocked by *V*_O_ defects in the light-field Cs-HRTEM image in the inset. We have added more data to verify the prevalence of domain defects with *V*_O_ in Cs-HRTEM images in Fig. S15. This indicates that a large number of oxygen vacancies are randomly distributed in the domain defects in the sample after the high-temperature treatment in a reducing gas. Although the *V*_O_ is isolated according to theory calculation, we still observe the slight agglomeration of *V*_O_ in lattice. However, the Ga_2_O_2.880_ still maintains the single-crystalline features, to well stabilize the *V*_O_ in the lattice.

To further understand the local structural differences of the PSC Ga_2_O_3_ monoliths with or without *V*_O_, systematic Ga *K*-edge extended x-ray absorption fine structure (EXAFS) data are studied by applying Athena-Artemis software in Fig. 2F and Figs. S16 and S17 [27–29]. We observe that there is no phase transformation with or without *V*_O_ in the PSC Ga_2_O_3_ monoliths, and the β-Ga_2_O_3_ phase is still identified [30,31]. It can be seen that the model well fits with the experimental data. The *R*-factor is <0.2% in the fitting results with all samples. Actually, the slight variation of coordination number is quite negligible for Ga–O and Ga–Ga of the samples even after reduction because the change of oxygen nonstoichiometry is quite small in Ga_2_O_3-δ_. Therefore, it is a usual way to fit the EXAFS data by fixing the coordination number of Ga–O in GaO_4_ tetrahedra and GaO_6_ octahedra at 4 and 6, respectively. All samples are likely to have 1 oscillation in the Ga–O shell. From the fit, the Ga–O bond length is gradually shortened from 1.882 to 1.747 Å for the samples after reduction. Moreover, it is observed that the Ga–Ga bond length is slightly shortened from 3.206 to 3.202 Å for the sample after reduction. We have added the O *K*-edge EXAFS of PSC Ga_2_O_3_ monoliths before and after reduction in Fig. S18. The O *K*-edge spectrum is divided into 2 regions. The first region is between 533 and 544 eV and possesses 2 broad absorption bands. Peaking at 537 and 542.8 eV, respectively, these 2 features arise mainly due to the excitation of O 2p–Ga 4s and O 2p–Ga 4p hybridization orbitals. These 2 polarized peaks tend to shift to lower energies with deeper reduction, and the peaks become shorter due to the shorter bond lengths of the Ga–O bonds. The second region is an EXAFS pseudo-oscillation produced by the multiple scattering effect of the O element with the surrounding metallic elements. This result indicates that *V*_O_ can exist stably in reduced PSC Ga_2_O_3_ monoliths, and these active structures are beneficial for improving dehydrogenation activity and carbon deposition resistance. In Fig. 2G to I and Fig. S19, the XPS results of the O 1s states of the PSC Ga_2_O_3_ monoliths after reduction indicates the changing trends of the concentration of *V*_O_ at surface. Three peaks of oxygen species, fitted from Gaussian simulation, can be attributed to lattice oxygen, oxygen vacancies, and adsorbed oxygen. The chemical state of Ga 2p remains basically unchanged of the 3 reduced PSC Ga_2_O_3_ monoliths. However, the binding energy of Ga 3d is 19.7 eV in XPS of PSC Ga_2_O_2.880_ monoliths after reduction. In contrast, the binding energy of Ga 3d is 19.5 eV in the XPS of PSC Ga_2_O_3_ monoliths before reduction. It can be seen that the binding energy of Ga 3d increases slightly with the increase of the concentration of oxygen defect in lattice. This suggests that the Ga–Ga bond is slightly strengthened, which is consistent with the EXAFS result.

**Fig. 3.**
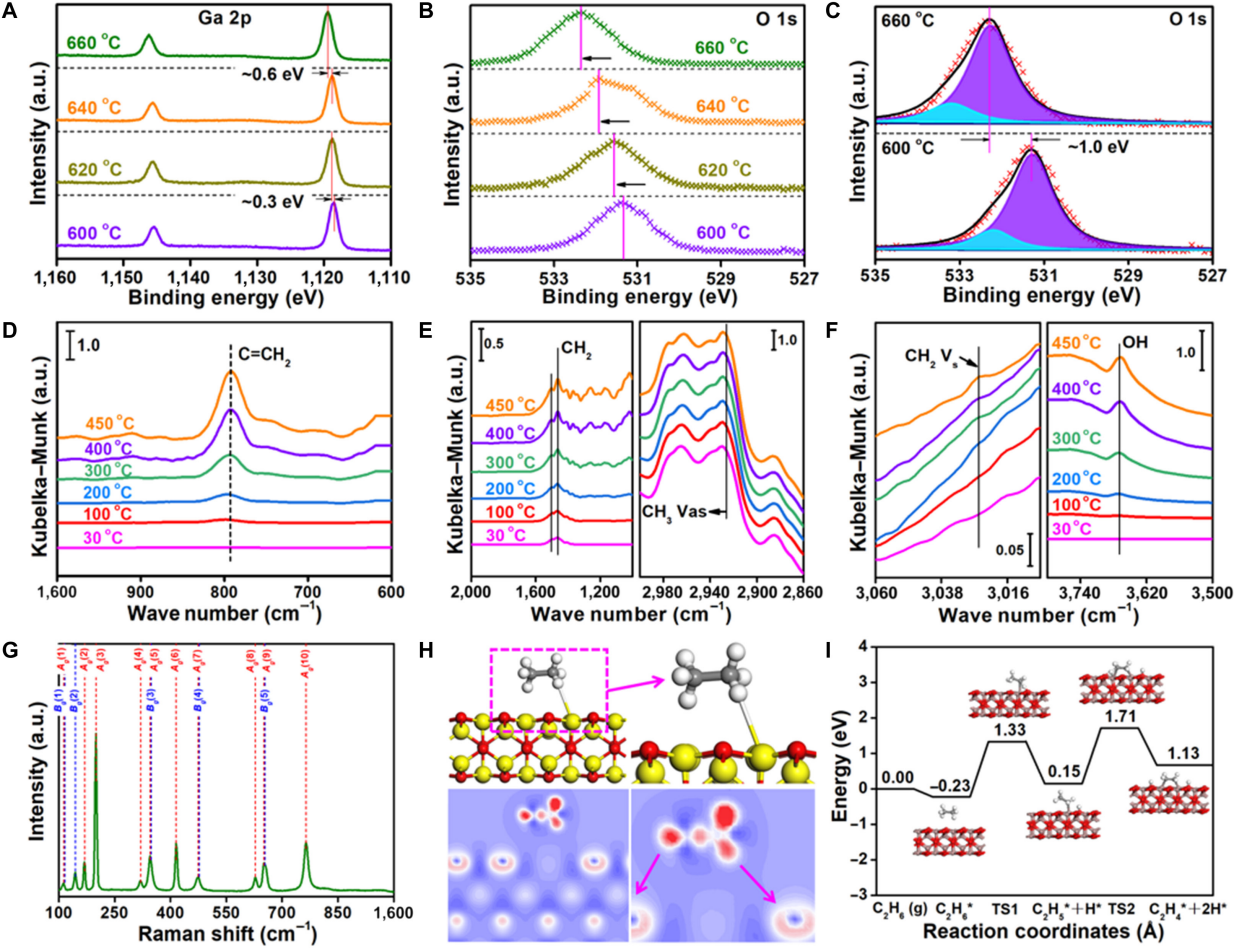
Mechanism of catalysis toward nonoxidative ethane dehydrogenation. (A to C) AP-XPS spectra of Ga 2p and O 1s under different temperatures. (D to F) In situ FTIR of the PSC Ga_2_O_2.880_ monoliths under different temperatures. (G) In situ Raman spectra of the PSC Ga_2_O_2.880_ monoliths at room temperature. (H) The activation process (top) and electron transfer (bottom) of β-Ga_2_O_3_ for ethane. (I) Potential energy of ethane dehydrogenation on β-Ga_2_O_3_ surface containing oxygen vacancies (gray is carbon, white is hydrogen, brown is gallium, and red is oxygen).

We further utilize ambient-pressure XPS (AP-XPS) in Shanghai Synchrotron Radiation Facility to analyze the chemical changes of Ga and O at surface in the PSC Ga_2_O_2.880_ monolith under reaction conditions [32,33]. Figure 3A to C shows the spectra of Ga 2p and O 1s of the catalyst in pure ethane from 600 to 660 °C. With increasing temperature, the binding energy of Ga 2p gradually increases by up to ~0.9 eV, indicating that the interaction force of the Ga–Ga bond is strengthened. The asymmetric peak of O 1s in Fig. 3C can be broken into 2 peaks by Gaussian fitting. The stronger peak is attributed to the oxygen in the reduced PSC Ga_2_O_3_, and the weaker peak is attributed to the C/O species adsorbed on the surface. The binding energy of O 1s is ~1.0 eV higher under a temperature range of 600 to 660 °C, indicating that the higher the temperature is, the easier it is to adsorb C_2_H_6_ molecules. We then utilize in situ Fourier transform infrared (FTIR) spectroscopy to survey the activation process of C_2_H_6_ in the PSC Ga_2_O_2.880_ monoliths in Fig. 3D to F. With increasing temperature, the asymmetric stretching vibration of the C–H bond in ethane is obviously shifted to the blue region, indicating that the C–H bond is effectively activated to a higher energy state. Additionally, the stretching vibrations of the C–H and C=C bonds of C_2_H_4_ become increasingly obvious, suggesting that the initial temperature of C_2_H_6_ dehydrogenation to C_2_H_4_ in the β-Ga_2_O_3_ phase is as low as ~200 °C. The CH_2_, C=CH_2_, and OH are the intermediates in the process of the nonoxidative dehydrogenation from C_2_H_6_ to C_2_H_4_ [34].

Information about the symmetry of the crystal structures is obtained by the Raman analysis, which indicates the monoclinic structure of β-Ga_2_O_3_. It consists of a tetrahedron structure (Ga_I_O_4_) and an octahedron structure (Ga_II_O_6_) and includes 2 categories of Ga atoms (Ga_I_ and Ga_II_) and 3 categories of O atoms (O_I_, O_II_ and O_III_) in Fig. S20. We utilize in situ Raman spectra to survey the phase transition of C_2_H_6_ in the PSC Ga_2_O_2.880_ monoliths in Fig. 3G. The Raman peak confirms that no phase transition occurred [35]. Figure S21 shows the FTIR spectroscopy of adsorbed pyridine for distinguishing between a Lewis acid and Bronsted acid. The absence of Bronsted acid in the β-Ga_2_O_3_ phase is indicated by the absence of a characteristic absorption band at ~1,540 cm^−1^. Figure 3H shows the configuration of chemisorption of C_2_H_6_ at surface, which indicates that the electrons are transferred from H to Ga atoms in the configurations. Figure 3I shows the process (C_2_H_6_ (g) → C_2_H_6_* → C_2_H_5_* + H* → C_2_H_4_* + 2H*) and the density functional theory (DFT)–calculated potential energy for the direct dehydrogenation of C_2_H_6_. The adsorption energy (Eads) of the C_2_H_6_ molecule is −0.23 eV, and the energy barrier is 1.56 eV for each elementary process. Reasonable activation energies and potential energy barriers indicate that the adsorbed C_2_H_6_ molecules can be efficiently activated, and the C–H bond cleavage tends to convert C_2_H_6_ to C_2_H_4_.

**Fig. 4.**
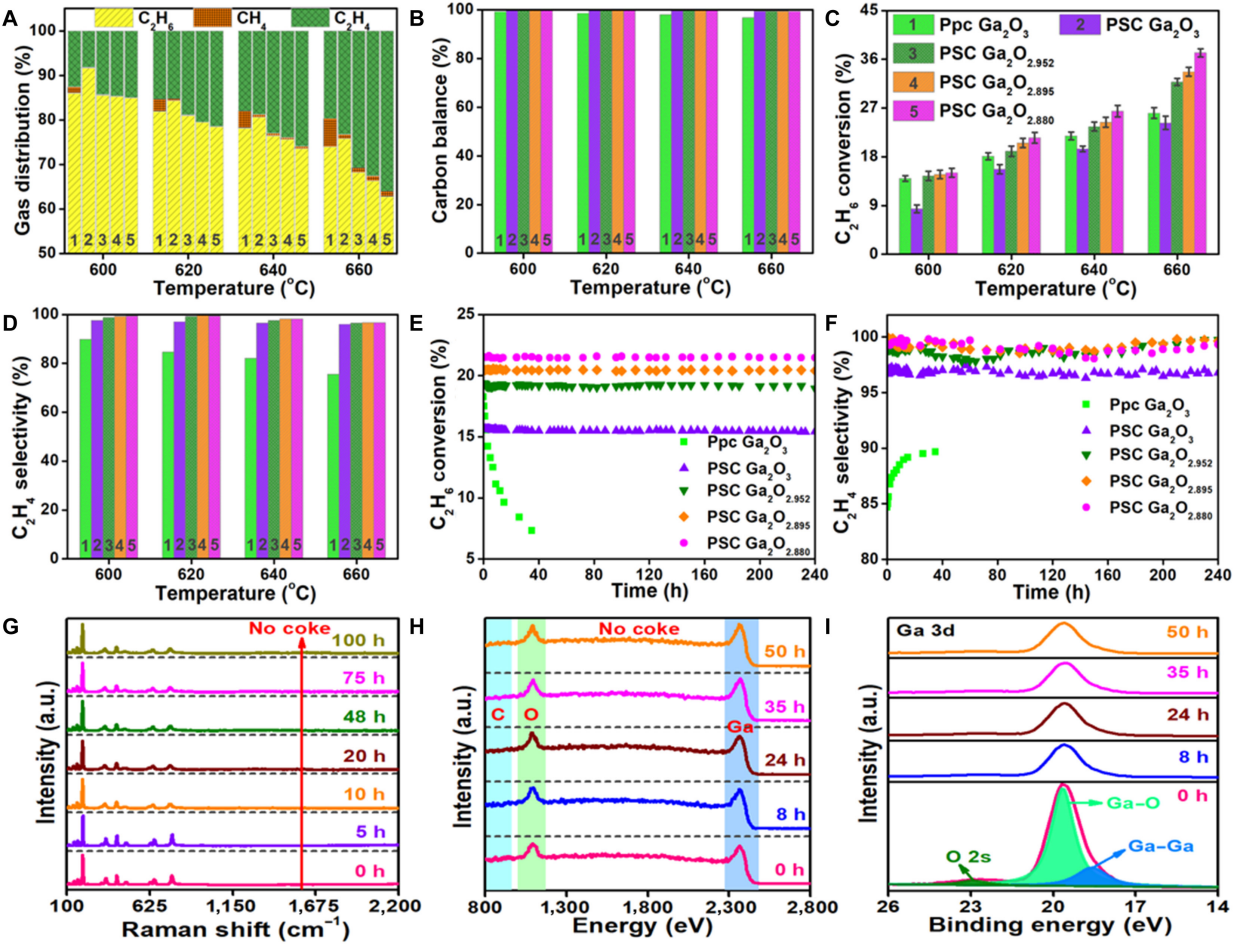
Nonoxidative ethane dehydrogenation to ethylene. (A to D) Product analysis, carbon balance, ethane conversion, and ethylene selectivity with different samples. (E and F) Long-term stability of ethane dehydrogenation at 620 °C. (G to I) In situ Raman, in situ HS-LEISS, and in situ XPS of the PSC Ga_2_O_2.880_ monoliths in pure ethane. Ppc Ga_2_O_3_ stands for porous polycrystalline β-Ga_2_O_3_.

Figure 4A and Fig. S22 display the product analysis for the nonoxidative dehydrogenation from C_2_H_6_ to C_2_H_4_. We discover that the ethylene is a dominant product and that the methane exists as a by-product. This result suggests a carbon balance of ~100% in Fig. 4B, further indicating that there is no carbon deposition during the reaction. The well-defined surfaces coupled with tailored *V*_O_ effectively improve the activation of C–H bonds in C_2_H_6_ and coking resistance. Figure 4C and D and Fig. S23 display the C_2_H_6_ conversion and C_2_H_4_ selectivity during the nonoxidative dehydrogenation process. C_2_H_6_ conversion on PSC Ga_2_O_2.880_ monoliths gradually increase versus temperature and finally reaches ~37% at 660 °C. In the control experiment, it is ~5 times higher than that of the thermal cracking of C_2_H_6_ without catalyst. The C_2_H_4_ selectivity of the PSC Ga_2_O_2.880_ monoliths can be up to ~100%. That of the control experiment is much higher than the ethylene selectivity of the thermal cracking of C_2_H_6_ without a catalyst. Here, the well-defined surfaces coupled with tailored *V*_O_ are considered to be active sites for the dehydrogenation of C_2_H_6_. The property of the active site governs the difference in the catalytic conversion and selectivity for dehydrogenating C_2_H_6_ to C_2_H_4_. In addition, the C_2_H_6_ dehydrogenation of different samples is performed at 620 °C, and stable C_2_H_6_ conversion and C_2_H_4_ selectivity are shown in Fig. 4E and F without degradation after 240 h of operation. We have added the characterization of the catalysts after the stability test for the dehydrogenation of C_2_H_6_ at 620 °C in Fig. S24. The XRD patterns confirm the structural stability of the 3 reduced PSC Ga_2_O_3_ monoliths after the stability test. The chemical states of Ga and O remain basically unchanged after the stability test. The Raman spectra confirm no visible coke formation after the stability test. In addition, the microstructure of the PSC Ga_2_O_2.880_ monoliths generally remain unchanged after stability test in Fig. S25. The domain defects associated with oxygen vacancies are not increased in lattice, which also reveals the excellent structural stability of PSC Ga_2_O_2.880_ monolith.

In order to further validate the stability, we conduct in situ spectroscopy to understand the surface states of the PSC Ga_2_O_2.880_ monoliths during the reaction. In Fig. 4G, we perform in situ Raman scattering studies of the PSC Ga_2_O_2.880_ monoliths from 0 to 100 h in pure C_2_H_6_ and find that the crystal structure remains stable without any visible change. In addition, we also find no obvious coke formation during the 100-h test. We perform in situ HS-LEISS spectra of the PSC Ga_2_O_2.880_ monoliths from 0 to 50 h in pure C_2_H_6_ in Fig. 4H. The compositions of Ga and O at the top layers remain essentially constant, and there is no carbon peak on the surface, indicating that the PSC Ga_2_O_2.880_ monoliths can exist stably under pure C_2_H_6_ at high temperature. These results indicate that the well-defined surfaces of the PSC Ga_2_O_3_ monoliths can accommodate oxygen vacancies, thus stabilizing the crystal structure and improving the carbon deposition resistance. Figure 4I presents the in situ XPS spectra of the PSC Ga_2_O_2.880_ monoliths after 0 to 50 h of reaction in pure C_2_H_6_. The binding energy peaks of Ga 3d at 22.96, 19.65, and 18.59 eV are visible, which originates from the O 2s, Ga–O, and Ga–Ga bonds of the reduced PSC Ga_2_O_3_, respectively. Moreover, the Ga 3d peak intensity is not visibly changed with increasing reaction time.

## Conclusion

In summary, we quantitatively introduce *V*_O_s and stabilize them by the long-range ordering of crystal lattice in a centimeter-scale PSC Ga_2_O_3_ monolith to boost the catalytic activity and durability. The oxygen vacancies are well stabilized in lattice even with slight agglomeration into domain defects ~1 to 2 nm at a high oxygen deficiency. The formation of low-coordinated Ga active sites therefore delivers an exceptionally high C_2_H_4_ selectivity of ~100% at an C_2_H_6_ conversion of ~37% even only at 660 °C for the nonoxidative dehydrogenation of C_2_H_6_ to C_2_H_4_. We have further demonstrated the outstanding stability in a continuous operation of up to 240 h. This work would not only facilitate the rational design of catalytic materials but also find potential applications of the porous single crystals in other fields.

## Materials and Methods

### Synthesis

We grew single-crystalline GaPO_4_ using the Bridgman method and cut it into (-132) and (012) substrates to grow porous single crystals. We treated the single-crystalline GaPO_4_ substrates at ~100 to 400 Torr (pure Ar) for 50 h at ~1,100 to 1,300 °C to grow PSC Ga_2_O_3_ monoliths. The flow rate of pure Ar was ~100 to 500 sccm, and then the crystals were slowly cooled to room temperature under a slow flow of pure Ar. We constructed oxygen vacancies in the PSC Ga_2_O_3_ monoliths by heat treatment with a reducing gas (5% H_2_/95% Ar) at 700 °C for ~5 to 20 h. The gas flow rate is 50 ml min^−1^.

### Characterization

The crystalline structure of samples was characterized by using XRD (Cu-Ka, Empyrean, Panalytical). The microstructures, morphologies, and elemental analysis of the samples were analyzed by using a field emission scanning electron microscope (FE-SEM) and EDS (SU-8010). TOF NPD was collected on the general-purpose powder diffractometer with a 360° rotation stage at China Spallation Neutron Source. The TOF NPD datasets were determined by Rietveld refinement performed on a crystallography data analysis software GSAS-II. Brunauer–Emmett–Teller test was conducted using a porosity analyzer (Tristar II 3020). The lattice structures, SAED patterns, and elemental mapping of the samples were analyzed using Cs-TEM (FEI Titan3 G2 60-300). Focused ion beam nanotomography (Hellos 650, Zeiss Auriga) was performed to obtain TEM slices with porous ultrathin microstructures. The composition of the top layer of the sample was examined by HS-LEISS (Otac100, ION-TOF). The Ga *K*-edge EXAFS was collected at the 21A x-ray nanodiffraction beamline of Taiwan Photon Source, National Synchrotron Radiation Research Centre, with photon fluxes on the sample ranging from 1 × 10^11^ to 3 × 10^9^ photons per second and x-ray energies of 6 to 27 KeV. The O *K*-edge EXAFS was executed by the 4W7B of Beijing Synchrotron Radiation Facility (BSRF). The operating voltage of the BSRF storage ring is 2.5 GeV, and the average current is 250 mA. The chemical states of elements were determined by XPS (ESCALAB 250Xi). Thermal gravimetric analysis was conducted using a Netzsch STA449F3 to calculate oxygen nonstoichiometry. Raman (HR800 Evolution, Horiba) was used to analyze the samples. ESR (E500, Bruker-BioSpin) was used to confirm the existence of oxygen vacancies. Near AP-XPS experiment was performed at the beamline BL02B01 of Shanghai Synchrotron Radiation Facility. In situ FTIR measurement was carried out using an IR spectrometer (Bruker VERTEX 70).

### Catalytic test

Catalytic tests were carried out under atmospheric pressure using the tube reaction system, which had a flow quartz microreactor with an inner diameter of 3 mm. For the dehydrogenation of C_2_H_6_, the gas reactants contained 10 vol% C_2_H_6_ and 90 vol% Ar. The flow rate of the gas reactant was 5 ml min^−1^. The reaction products were analyzed using an online gas chromatograph (GC2014, Shimazu, Japan) equipped with a flame ionization detector (FID) and a thermal conductivity detector (TCD) and a 30-m packed column of CP-poraplot Q.

### Theoretical calculations

Spin-polarized DFT calculations were carried out a periodic slab model using Vienna Ab initio Simulation Package. The electron–ion interaction was clearly defined by the projected augmented wave pseudopotential with a plane wave cutoff of 500 eV. The generalized gradient approximation, including Perdew–Burke–Ernzerhof, was applied to display the exchange and correlation functional throughout the calculations, which provided an efficient method for calculating the energies of all intermediates and transition states (TSs). During geometry optimization, 10^−5^ eV and 0.01 eV Å^−1^ were used for convergence of the energies and residual forces. The β-Ga_2_O_3_ unit cell was calculated on a 3 × 10 × 6 k-point grid, and the optimized lattice parameters were *a* = 12.502 Å, *b* = 3.099 Å, and *c* = 5.912 Å. To avoid the low-layer interaction with the periodic image, a vacuum gap of 20 Å was set up for a p (4 × 2) superstructure of the β-Ga_2_O_3_ (100) surface, where the bottom 5 layers of the atoms were frozen, the top 5 layers were fully relaxed, and a 3 × 3 × 1 k-point grid was applied for Brillouin zone sampling. We used the climbing image nudged elastic band method to calculate the minimum reaction path for the C−H bond cleavage process and obtained the initial TS structure, which was subsequently used in Henkelman’s dimer method to identify the final TS, and each TS had only 1 imaginary frequency. The adsorption energy and activation barrier were calculated by *E*_ads_ = E_adsorbate/sur_ − *E*_adsorbate_ − *E*_sur_, *E*_T_ = *E*_TS_ − *E*_IS_, where *E*_adsorbate/sur_ is the total energy of surface and adsorbed ethane, *E*_adsorbate_ is the energy of a single ethane molecule, *E*_sur_ is the energy of the free surface, *E*_TS_ is the energy of the TS structure, and *E*_IS_ is the energy of the initial state of the reactant.

## Data Availability

The data used to support the findings of this study are available from the corresponding author upon request.
