# Supplementary material for: Insight into the Role and Evidence of Oxygen Vacancies in Porous Single-Crystalline Oxide to Enhance Catalytic Activity and Durability
Source: Research (Wash D C). 2023 Sep 21;6:0233. doi: 10.34133/research.0233 (PMC11776074; doi:10.34133/research.0233)
Supplement: Supplementary 1 — Figs. S1 to S25 [file research.0233.f1.docx]

**Supporting Information**

**Insight into the Role and Evidence of Oxygen Vacancies in Porous Single-Crystalline Oxide to Enhance Catalytic Activity and Durability**

Lingting Ye,^1,2,3^ Jiaming Ma,^1,2,3^ Jie Zhang,^1,2,3^ Wen Yin,^4^ Yuanguang Xia,^4^ Kui Xie^1,2,3,*^

^1^ Key Laboratory of Optoelectronic Materials Chemistry and Physics, Fujian Institute of Research on the Structure of Matter, Chinese Academy of Sciences, Fuzhou, Fujian 350002, China.

^2^ Fujian Science & Technology Innovation Laboratory for Optoelectronic Information of China, Fuzhou, Fujian 350108, China.

^3^ Advanced Energy Science and Technology Guangdong Laboratory, 29 Sanxin North Road, Huizhou, Guangdong 116023, China.

^4^ Spallation Neutron Source Science Center, Dongguan, Guangdong 523803, China.

* Corresponding Author’s E-mail: [kxie@fjirsm.ac.cn](mailto:kxie@fjirsm.ac.cn)

**Supplementary Figures.**


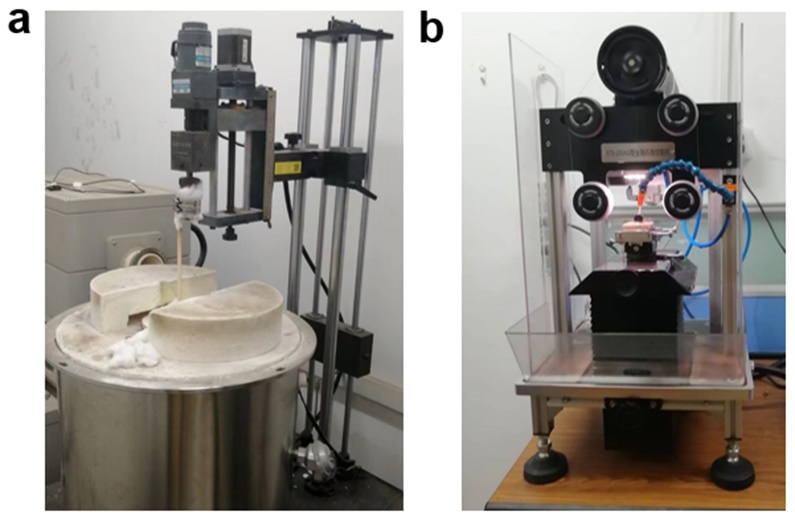


**Figure S1. Equipment for growing and cutting SC GaPO_4_ monoliths.** (a) The growth of SC GaPO_4_ monoliths. (b) The cutting of SC GaPO_4_ monoliths.


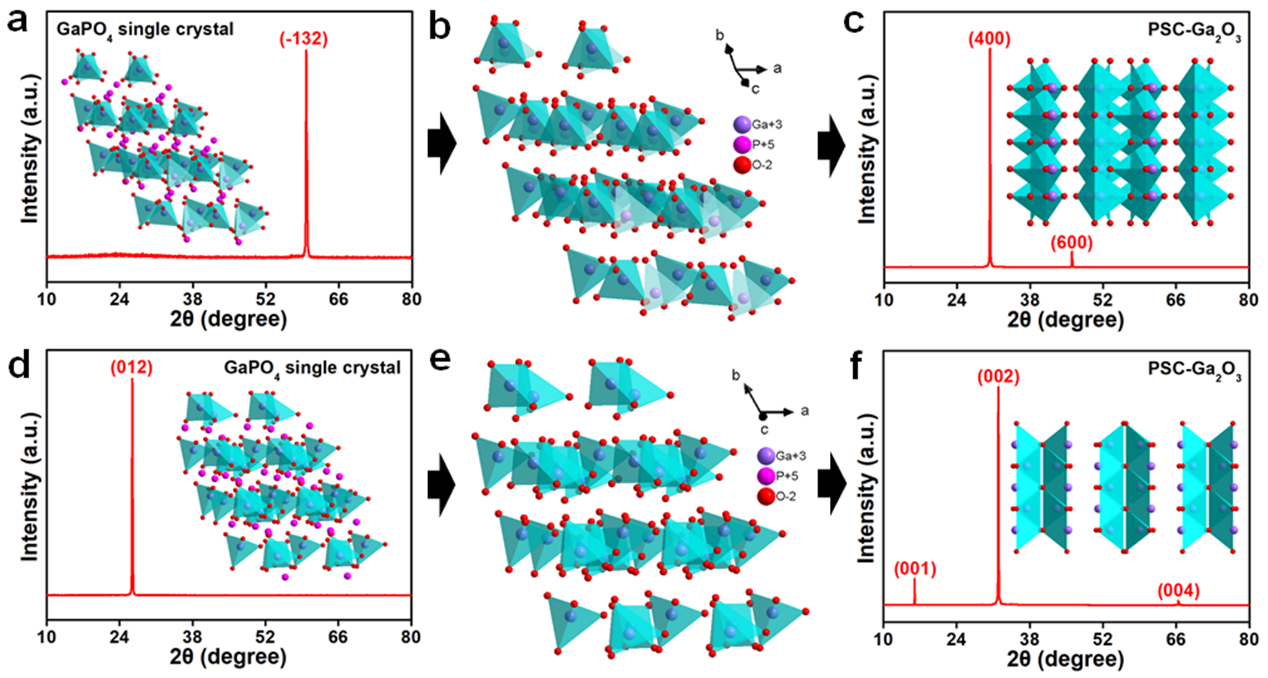


**Figure S2. Growth mechanism of PSC Ga_2_O_3_ monoliths from SC GaPO_4_ monoliths.** (a-c) The lattice reconstruction strategy of SC (-132) GaPO_4_ monoliths to the growth of PSC (100) Ga_2_O_3_ monoliths. (d-f) The lattice reconstruction strategy of SC (012) GaPO_4_ monoliths to the growth of PSC (001) Ga_2_O_3_ monoliths.


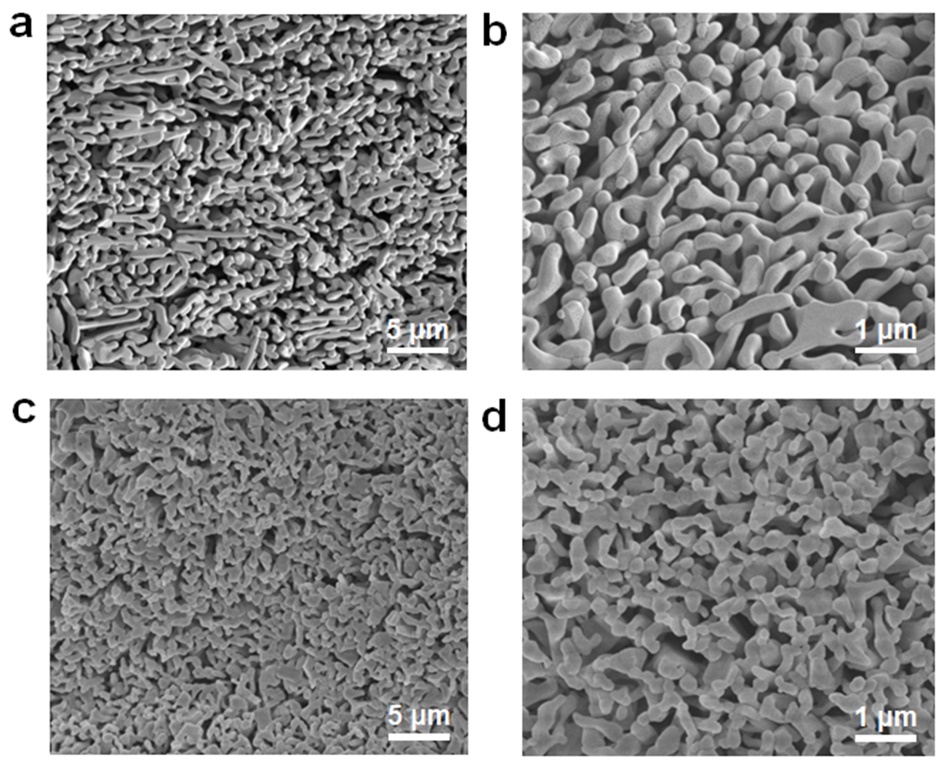


**Figure S3.** **The microstructure of PSC Ga_2_O_3_ monoliths.** (a-b) SEM images of PSC (100) Ga_2_O_3_ monoliths. (c-d) SEM images of PSC (001) Ga_2_O_3_ monoliths.


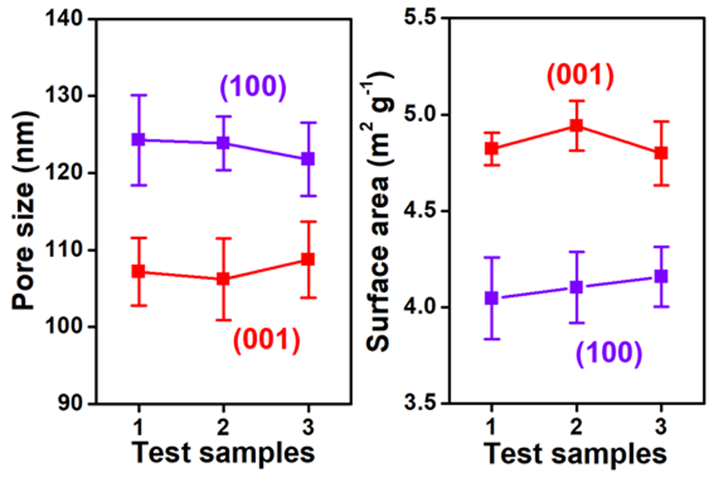


**Figure S4. The specific surface area and pore size of PSC Ga_2_O_3_ monoliths**. The BET pore size and surface area of the PSC (100) and (001) Ga_2_O_3_ monoliths.


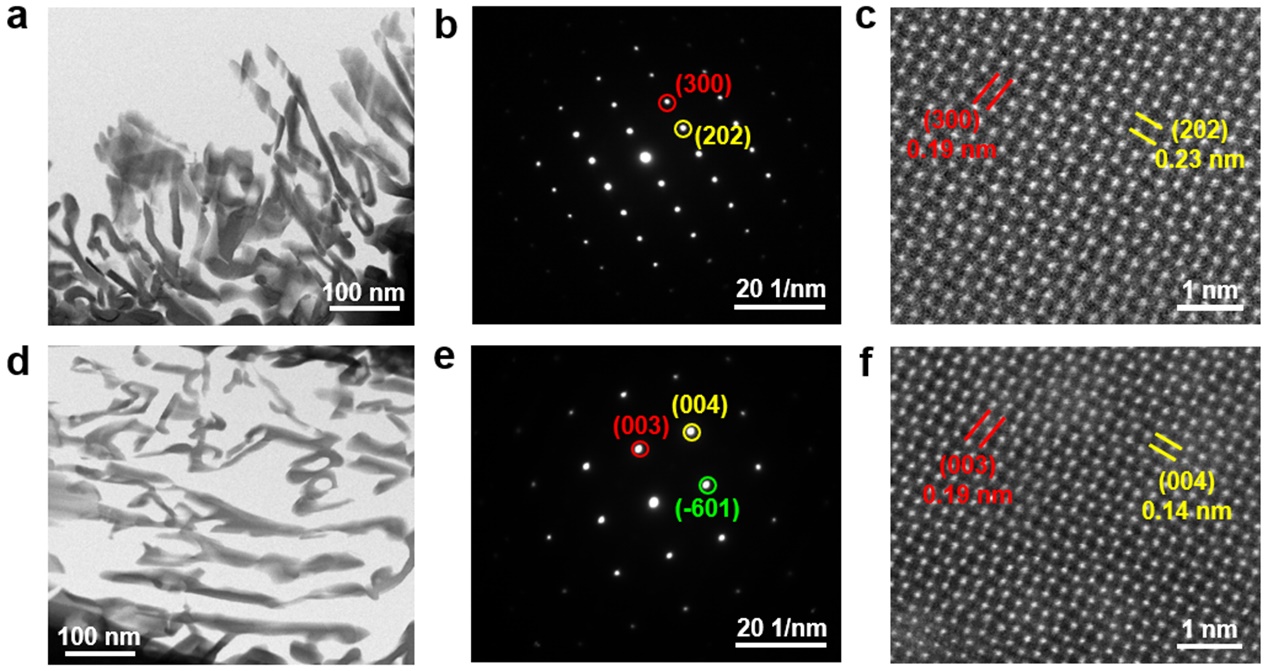


**Figure S5. Microstructure of PSC Ga_2_O_3_ monoliths.** (a-c) STEM, SAED and Cs-HRTEM of PSC (100) Ga_2_O_3_ monoliths. (d-f) STEM, SAED and Cs-HRTEM of PSC (001) Ga_2_O_3_ monoliths.


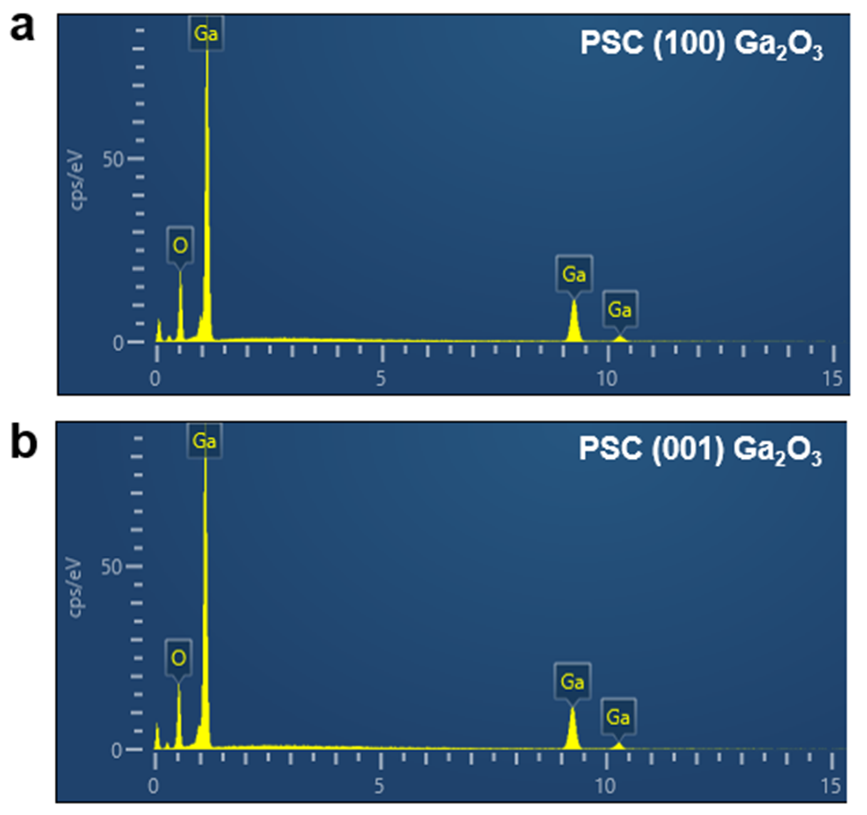


**Figure S6.** **The element analysis of PSC Ga_2_O_3_ monoliths.** (a) The EDS element analysis of PSC (100) Ga_2_O_3_ monoliths. (b) The EDS element analysis of PSC (001) Ga_2_O_3_ monoliths.


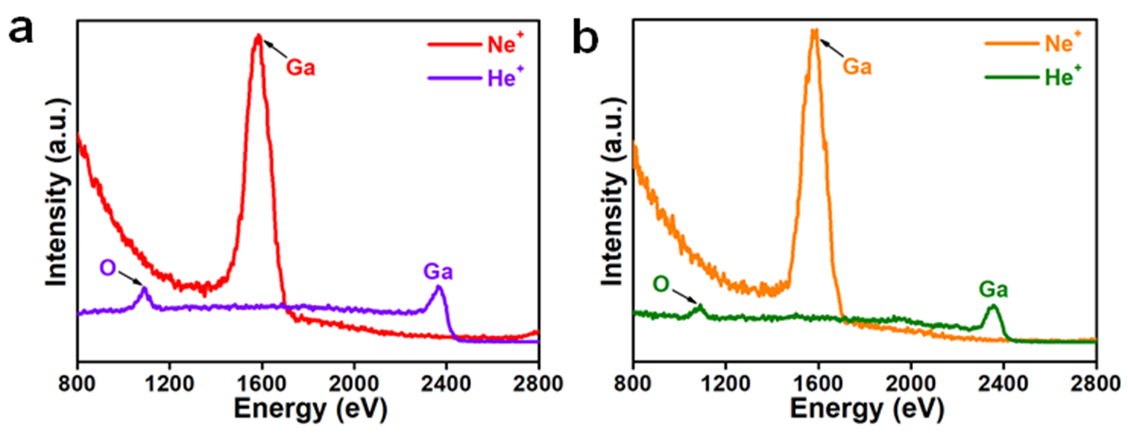


**Figure S7. The atomic termination layer of PSC Ga_2_O_3_ monoliths.** (a) The HS-LEISS of PSC (100) Ga_2_O_3_ monoliths. (b) The HS-LEISS of PSC (001) Ga_2_O_3_ monoliths.


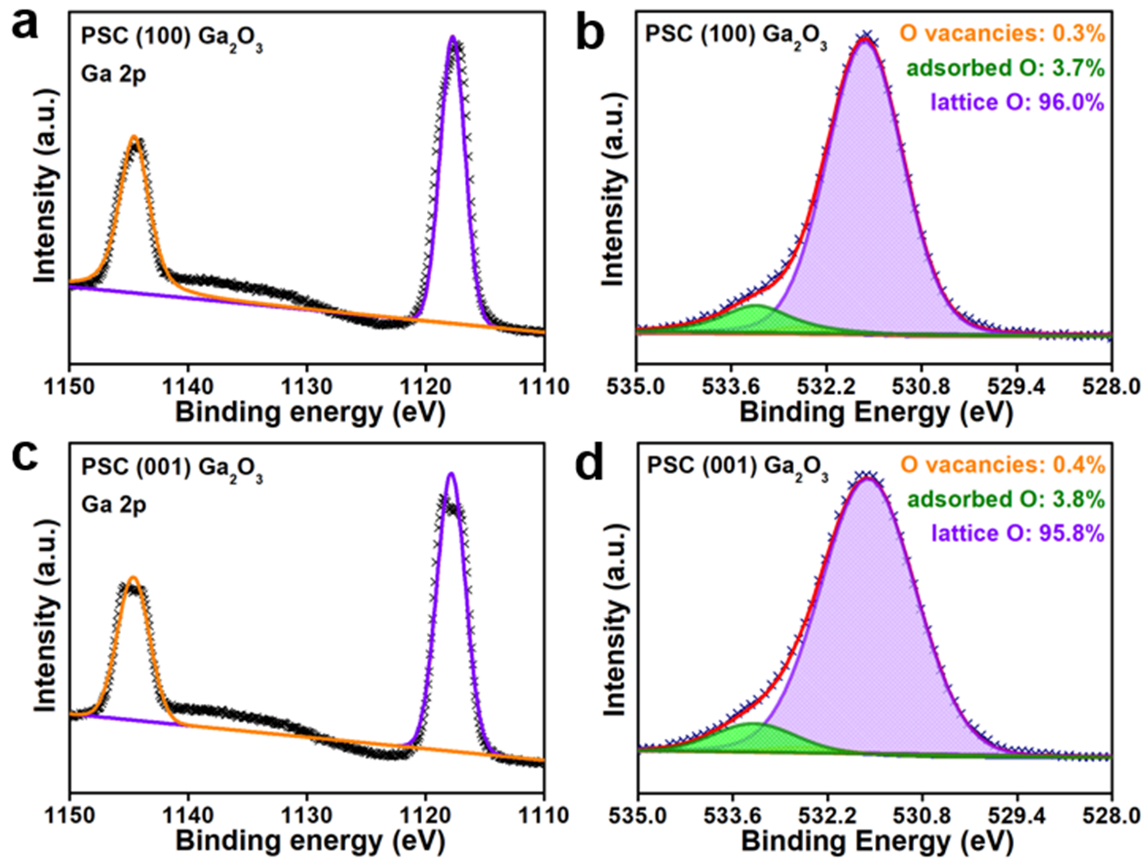


**Figure S8. The chemical state of PSC Ga_2_O_3_ monoliths.** (a-b) The XPS of Ga 2p and O 1s in PSC (100) Ga_2_O_3_ monoliths. (c-d) The XPS of Ga 2p and O 1s in PSC (001) Ga_2_O_3_ monoliths.


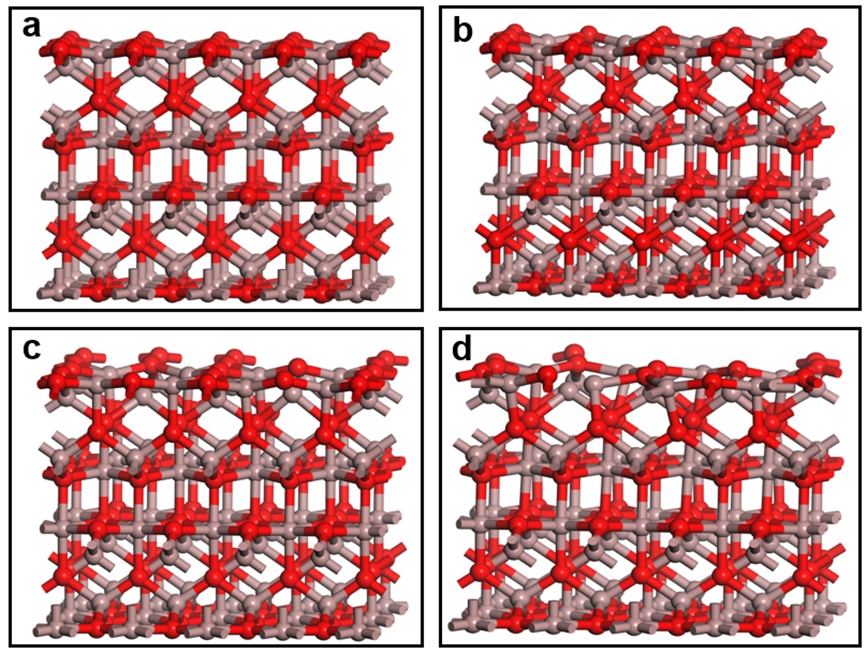


**Figure S9.** **Structure of β-Ga_2_O_3_ with different concentrations of oxygen vacancies.** (a) Perfect; (b) 2% Odef; (c) 4% Odef; (d) 6% Odef.


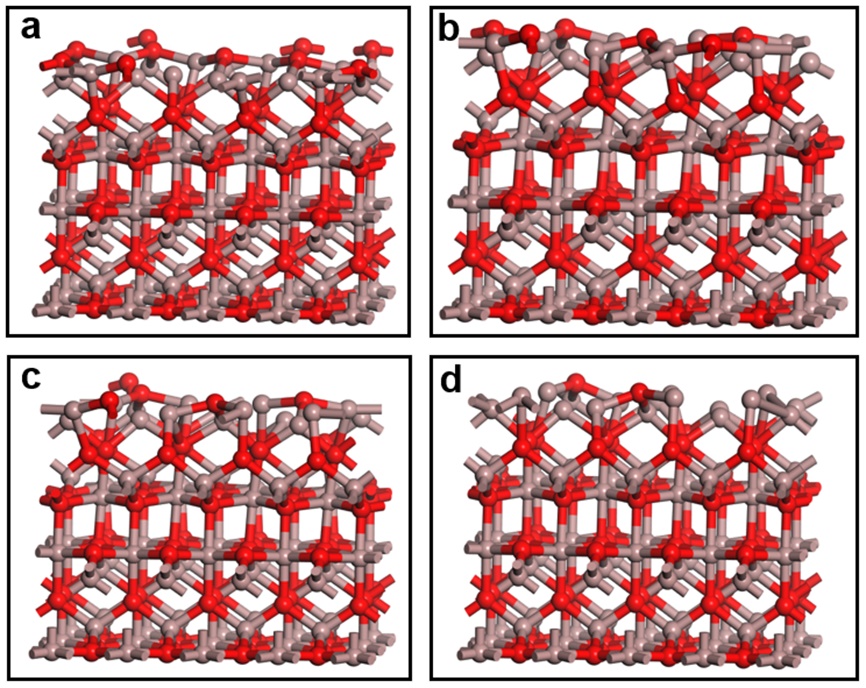


**Figure S10. Structure of β-Ga_2_O_3_ with different concentrations of oxygen vacancies.** (a) 8% Odef; (b) 10% Odef; (c) 12% Odef; (d) 14% Odef.


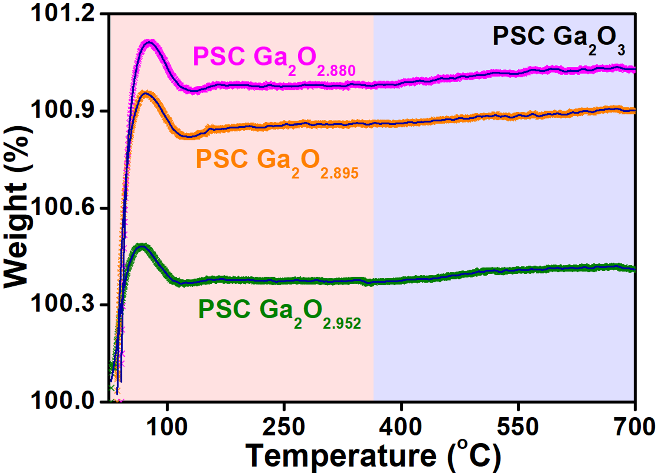


**Figure S11. Determine the chemical formula.** TGA tests of the three reduced PSC Ga_2_O_3_ monoliths in air atmosphere.


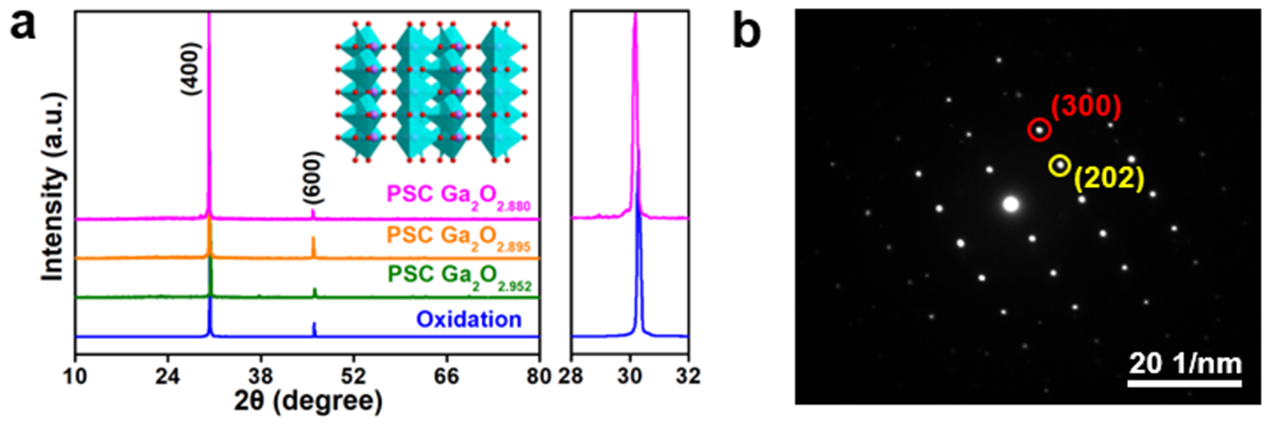


**Figure S12. The crystal structure of** **PSC Ga_2_O_3_ monoliths** **before and after reduction.** (a) The XRD patterns of PSC (100) Ga_2_O_3_ monoliths. (b) SAED of PSC (100) Ga_2_O_2.880_ monoliths.


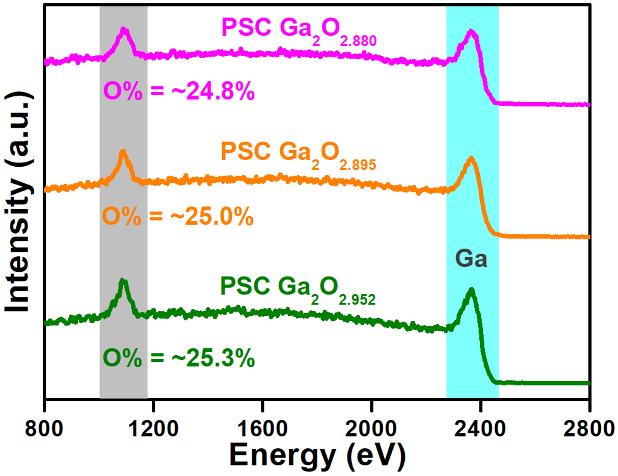


**Figure S13. Identify the ratio between O and Ga.** HS-LEISS spectra of the three reduced PSC Ga_2_O_3_ monoliths.


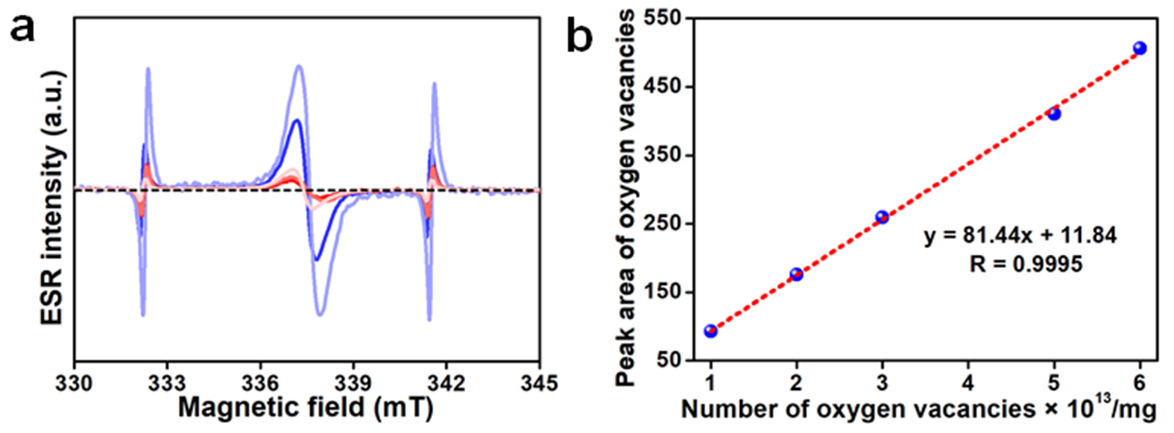


**Figure S14. Standard curve of ESR tests.** (a) ESR spectra of DPPH samples with different oxygen vacancies concentrations. (b) The standard working curve for the number of oxygen vacancies.


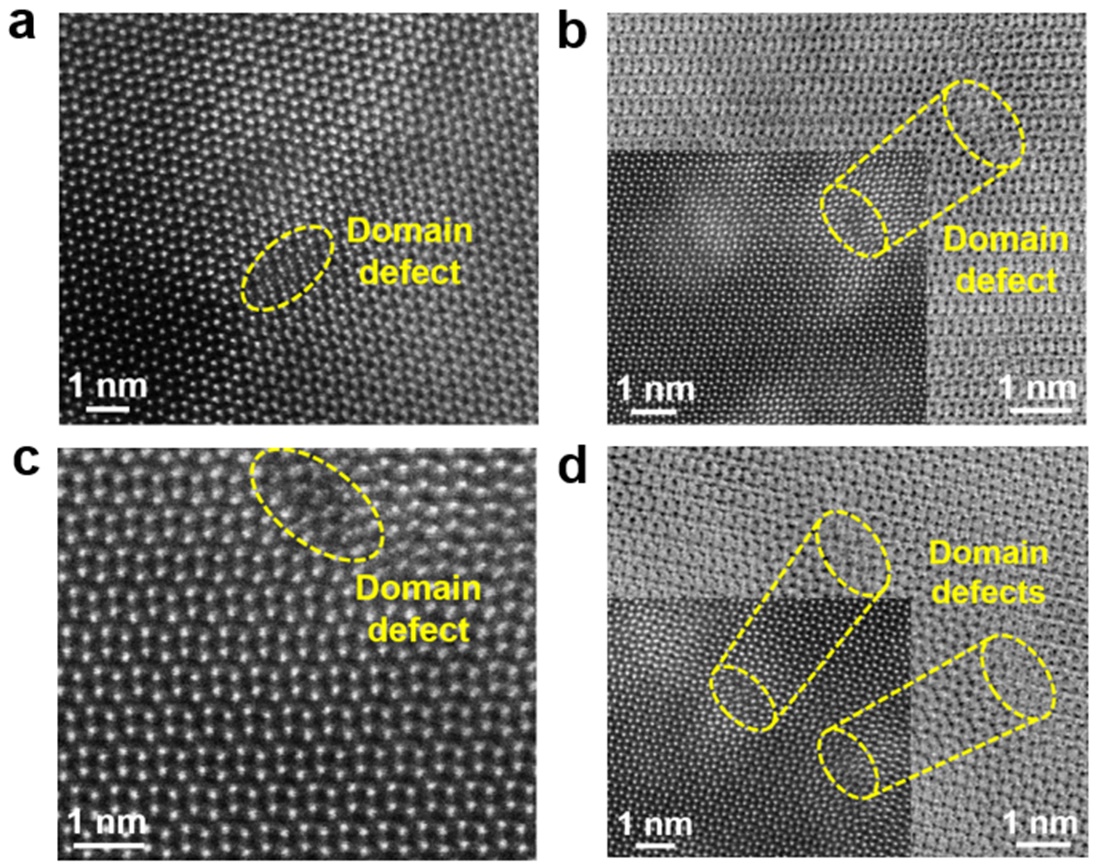


**Figure S15. The microstructure of PSC Ga_2_O_2.880_ monoliths.** (a-d) Cs-HRTEM images with different area of the PSC Ga_2_O_2.880_ monoliths.


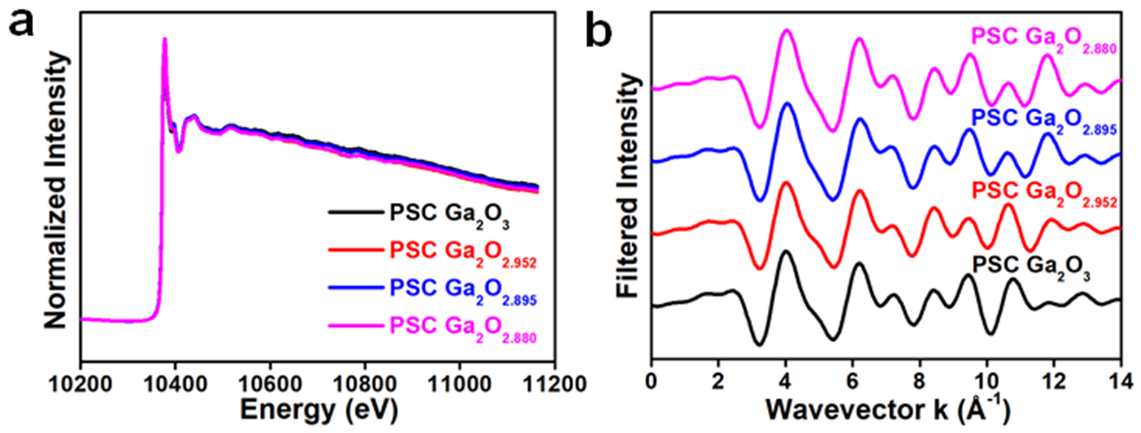


**Figure S16. Fine structure differences with or without oxygen vacancies in PSC Ga_2_O_3_ monoliths.** (a) Normalized Ga *K*-edge EXAFS of with different samples. (b) EXAFS oscillation as a function of wavevector, extracted from the EXAFS spectra.


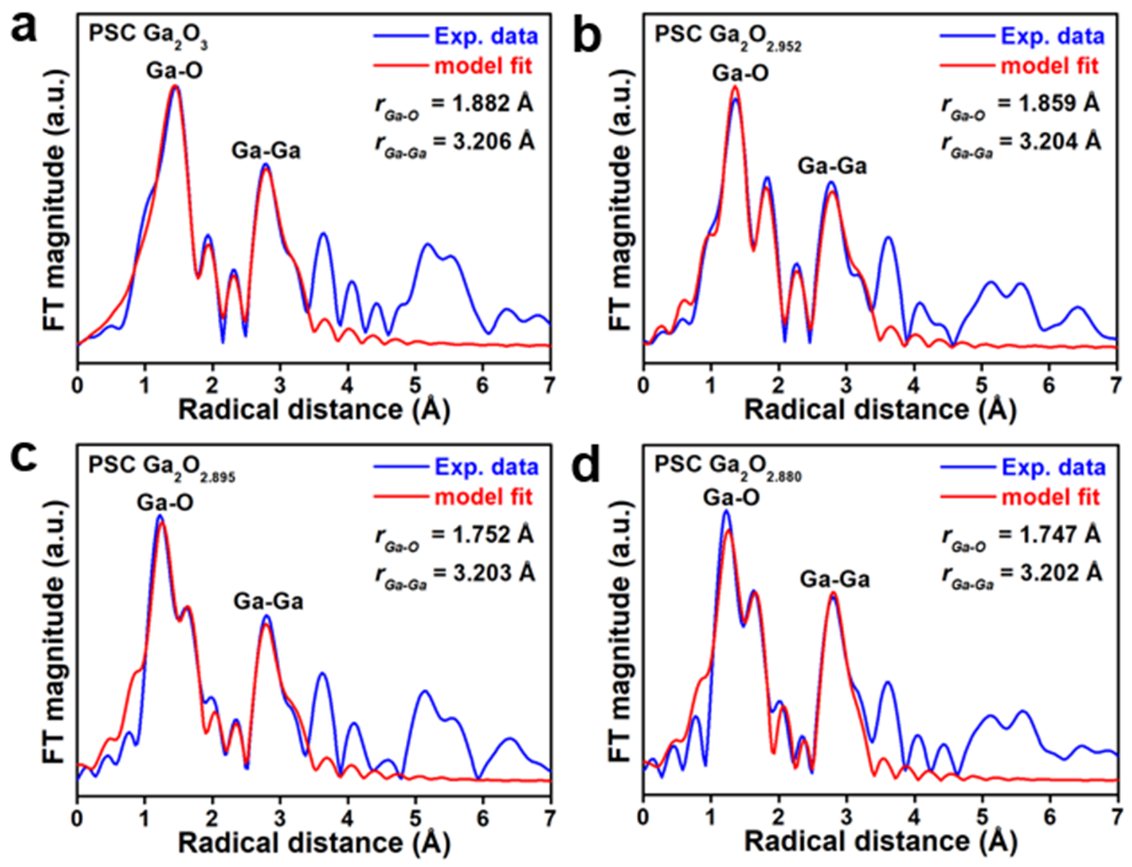


**Figure S17. The local structure differences of** **PSC Ga_2_O_3_ monoliths** **before and after reduction.** Fourier transforms of the Ga *K*-edge EXAFS spectra for the (a) PSC Ga_2_O_3_ monoliths, (b) PSC Ga_2_O_2.952_ monoliths, (c) PSC Ga_2_O_2.895_ and (d) PSC Ga_2_O_2.880_ monoliths.


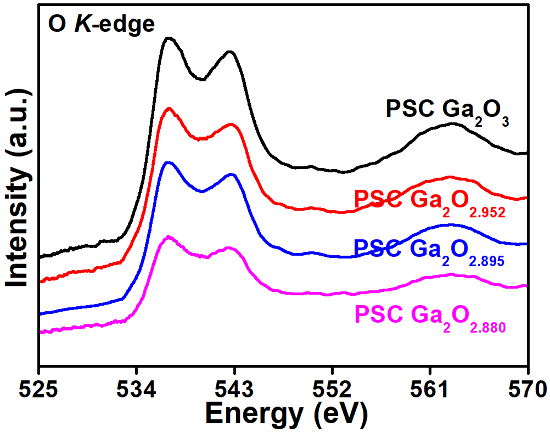


**Figure S18.** The O *K*-edge EXAFS of PSC Ga_2_O_3_ monoliths before and after reduction.


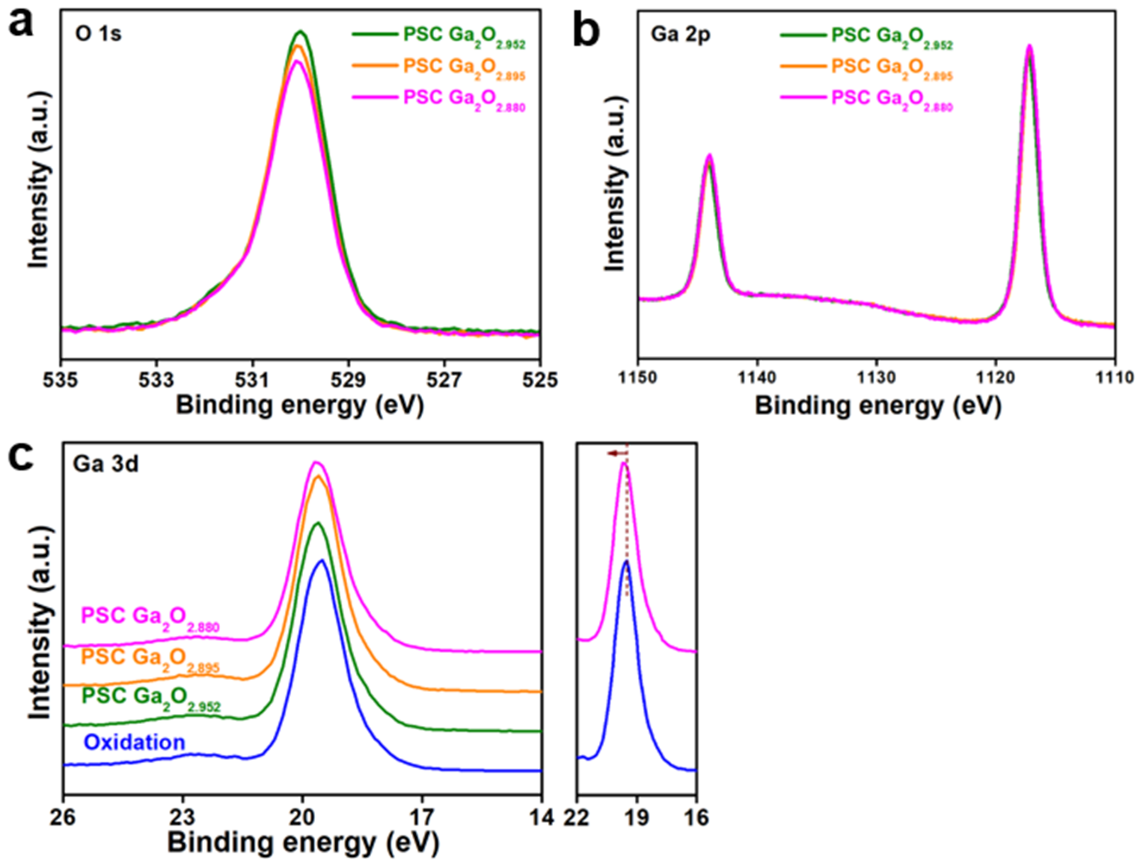


**Figure S19. The** **chemical state of PSC Ga_2_O_3_ monoliths after reduction.** (a-c) The XPS of O 1s, Ga 2p and Ga 3d in the three reduced PSC Ga_2_O_3_ monoliths.


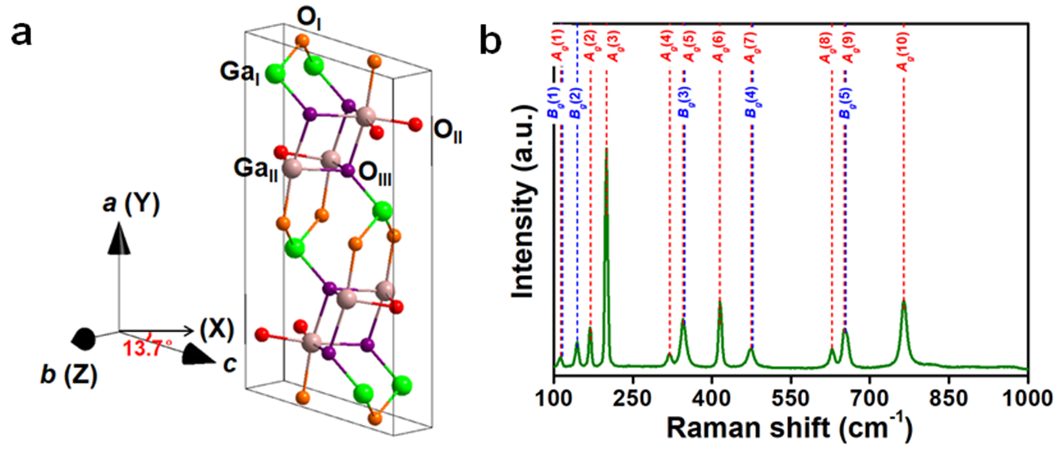


**Figure S20. Relationship between crystal structure and Raman spectra.** (a) Crystal structure of β-Ga_2_O_3_ with a unit cell. (d) *In situ* Raman spectra of the PSC Ga_2_O_2.880_ monoliths at room temperature.


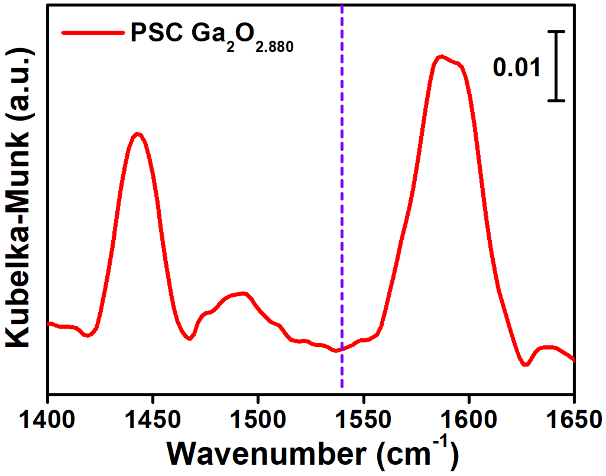


**Figure S21. Identify Lewis acid and Bronsted acid.** The FTIR spectroscopy of adsorbed pyridine of the PSC Ga_2_O_2.880_ monoliths at room temperature.


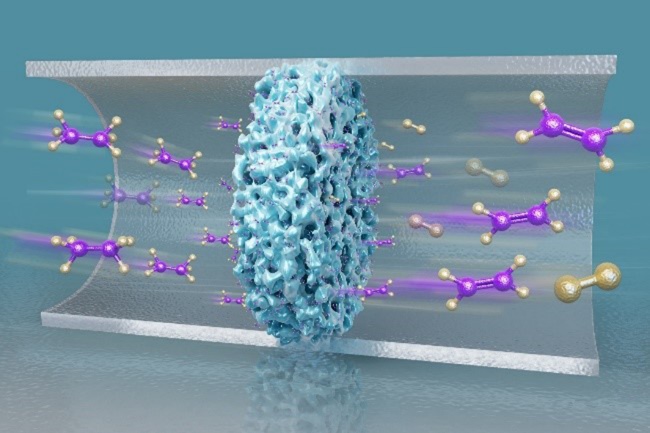


**Figure S22. Schematic diagram.** Non-oxidative ethane dehydrogenation to ethylene.


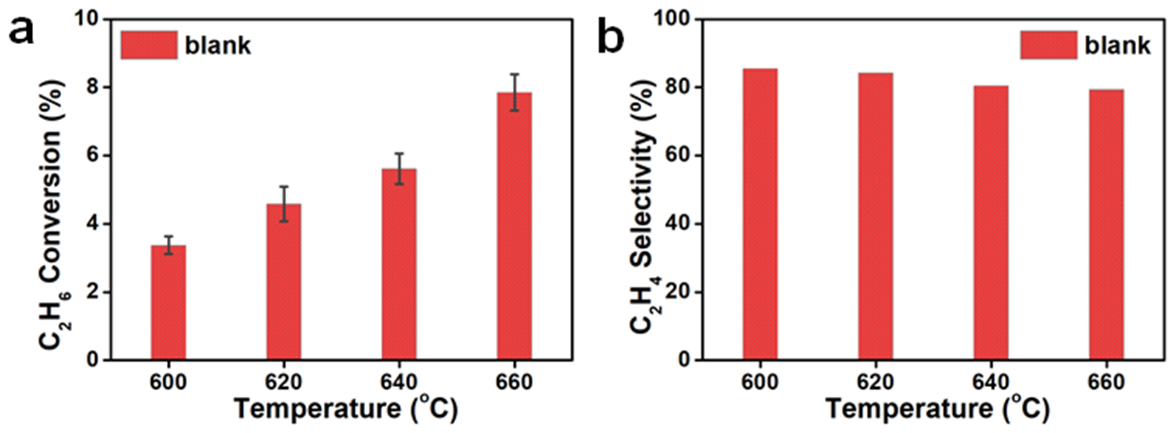


**Figure S23. Thermal cracking of ethane without catalyst in blank experiment.** (a) Ethane conversion and (b) ethylene selectivity from 600 ℃ to 660 ℃.


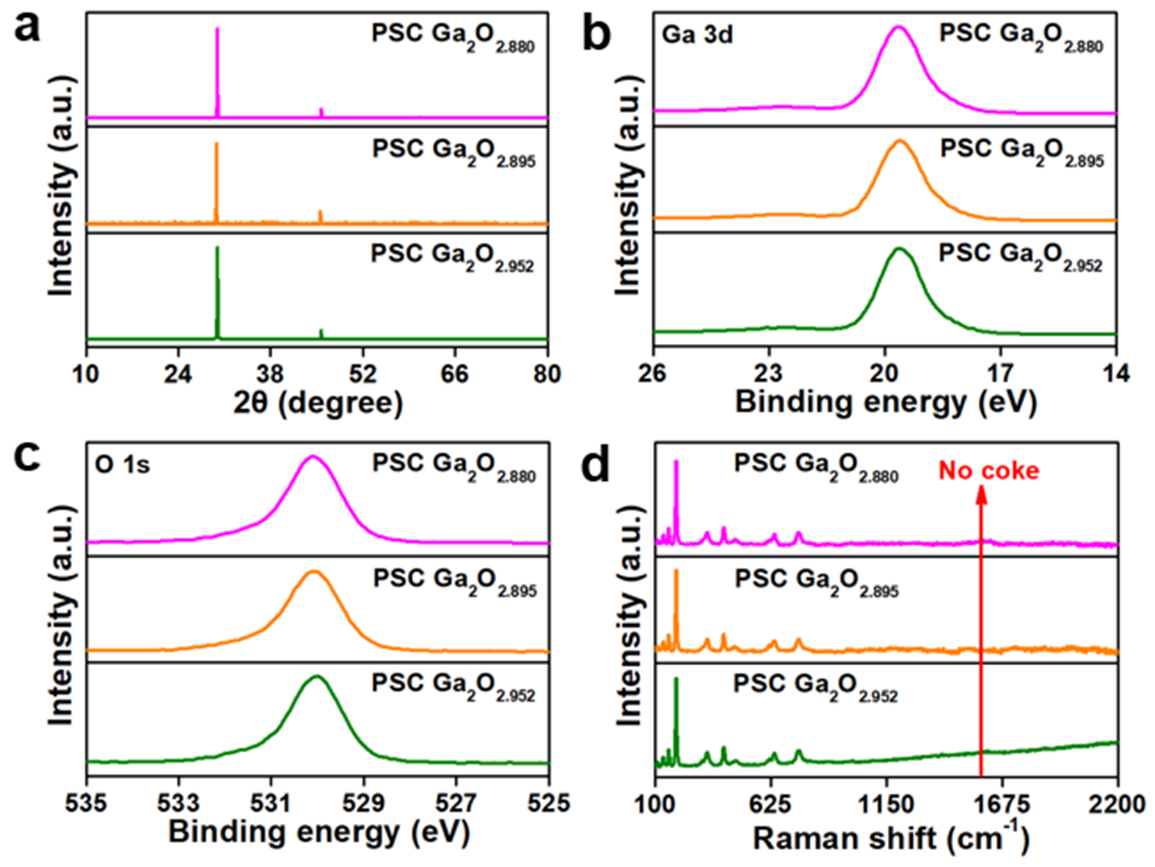


**Figure S24. The characterization of the three reduced PSC Ga_2_O_3_ monoliths after stability test.** (a) The XRD patterns after stability test. (b-c) The XPS of Ga 3d and O 1s after stability test. (d) The Raman spectrums after stability test.


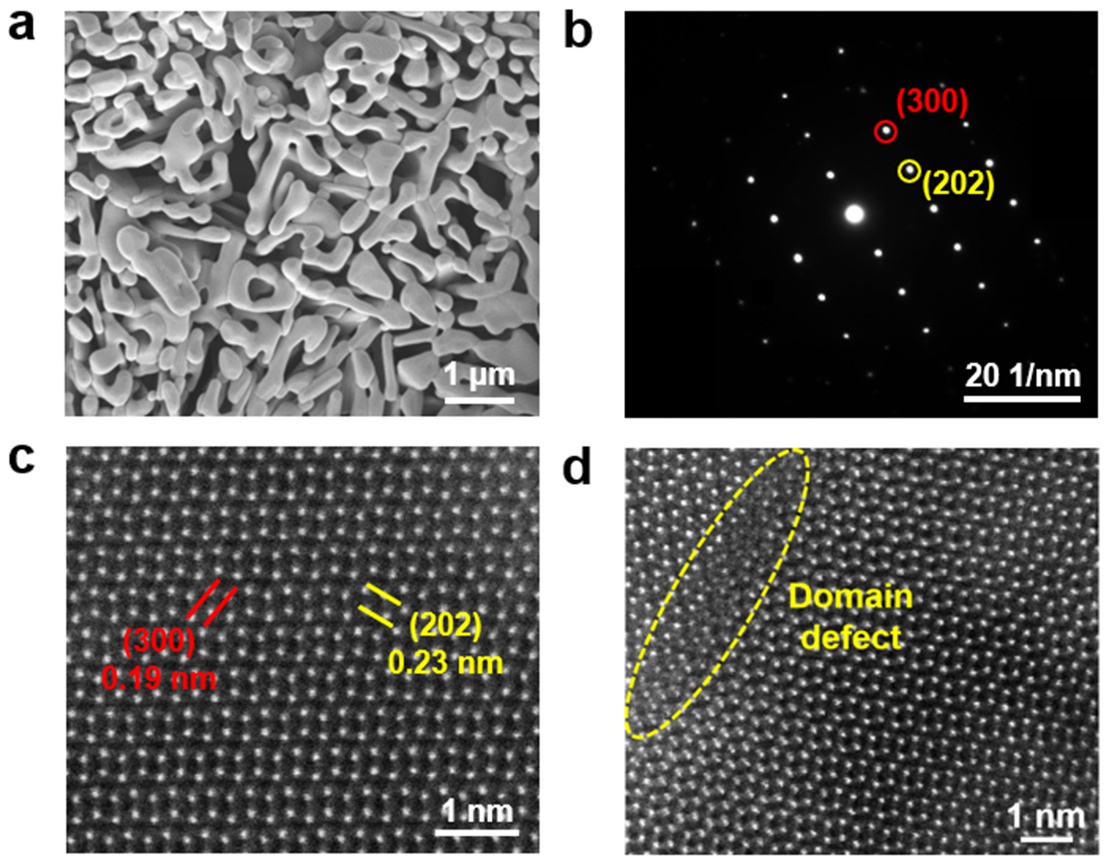


**Figure S25. The microstructure of** **PSC Ga_2_O_2.880_ monoliths after stability test.** (a-c) SEM, SAED and Cs-HRTEM of PSC Ga_2_O_2.880_ monoliths after stability test. (d) Cs-HRTEM with domain defect after stability test.
